# Compliance with the breakthrough cancer pain European guidelines and impact on patients' quality of life: an observational prospective study

**DOI:** 10.3389/fpain.2024.1388837

**Published:** 2024-06-28

**Authors:** Paolo Bossi, Tatiana Pietrzyńska, César Margarit Ferri, Irene Mansilla, Valeria Tellone, Sara Fioravanti, Giorgio Di Loreto, Alessandro Comandini

**Affiliations:** ^1^ Department of Biomedical Sciences, Humanitas University, Milan, Italy; ^2^ Medical Oncology and Hematology Unit, IRCCS Humanitas Research Hospital, Milan, Italy; ^3^ Palliative Care Ward, Czeladz Hospital, Czeladz, Poland; ^4^ Palliative Care Association “Hope”, Bedzin, Poland; ^5^ Home Hospice “Panaceum”, Dabrowa Gornicza, Poland; ^6^ Pain Unit, General University Hospital of Alicante, Alicante, Spain; ^7^ Medical Writing Department, TFS Health Science, Barcelona, Spain; ^8^ Global Medical Department, Angelini Pharma S.p.A., Rome, Italy; ^9^ Pharmacometrics & Clinical Supply, Angelini Pharma S.p.A., Rome, Italy

**Keywords:** breakthrough cancer pain, guidelines compliance, rapid-onset opioids, quality of life, BTcP management

## Abstract

**Introduction:**

This study aimed to assess the percentage of patients treated according to the European Society for Medical Oncology (ESMO) 2018 guidelines for breakthrough cancer pain (BTcP) and the impact of guidelines adherence on patients' quality of life (QoL).

**Methods:**

Adult opioid-tolerant patients diagnosed with BTcP and locally advanced or recurrent metastatic cancer with a life expectancy of >3 months prospectively were included. Patients were followed up for 28 days.

**Results:**

Of 127 patients included, 37 were excluded due to the impossibility to establish adherence to the ESMO guidelines. Among the evaluable patients [51.1% female; with mean (SD) age of 66.4 (11.8) years], all were adherent. BTcP was diagnosed by the Association for Palliative Medicine algorithm in 47.8% of patients and by clinical experience in 52.2% of patients. The mean number of daily BTcP episodes ranged between 1 and 8, with a mean (95% CI) severity of 7.3 (7.0; 7.6) at week 0 and 6.2 (5.8; 6.6) at week 4. Time to maximum pain intensity was 3–15 min in 52.2% of patients, and BTcP lasted 30–60 min in 14.4% of patients at week 0 and 4.4% of patients at week 4. Mean (95% CI) treatment effectiveness was 6.6 (6.1; 7.1) at week 0 and 7.4 (7.0; 7.8) at week 4. Median (Q1–Q3) patients' global impression of clinical condition was 4.0 (4.0–4.0) at week 0 and 3.0 (2.0–3.0) at week 4.

**Conclusion:**

A clear BTcP assessment and strict follow-up could be crucial to guidelines adherence and for patient's QoL.

## 1 Introduction

Breakthrough cancer pain (BTcP) is an episode of severe pain that occurs in cancer patients with stable and adequately controlled background pain and requires careful assessment and appropriate management (1). BTcP has an overall pooled prevalence of 59% (2) and typical BTcP episodes are of moderate to severe intensity, rapid onset (brief time to maximum pain intensity between 3 and 15 min) (3, 4), and short duration (15–30 min/episode or even shorter) (1, 3). The frequency of BTcP episodes varies from 3 to 6 daily episodes to several times a week (3–5). Moreover, BTcP negatively influences physical and mental health (5, 6), significantly increasing depression and anxiety (4) and functional impairment (7).

The lack of a universally accepted definition, classification, and validated clinical assessment tools, makes BTcP management difficult (1, 8). For the diagnosis of BTcP, the Association for Palliative Medicine (APM) algorithm (also called Davies algorithm) (9), continues to be widely used in practice (1, 10), whereas the Assessment Tool-BAT (11) was validated to facilitate diagnosis, management, and periodic monitoring of BTcP patients. Moreover, conventional BTcP treatment is often suboptimum (12), since it includes oral morphine and other normal-release oral opioids, which show pharmacokinetics and pharmacodynamics that do not match the characteristics of most BTcP episodes, resulting in a delayed effect or ineffectiveness (3). Nevertheless, the ideal characteristics of a BTcP drug are: potent analgesia, rapid onset of action, short-lasting effect, minimum side effects, and easy administration (the parenteral route is not always possible in a home setting) (12). Furthermore, BTcP management and treatment can be unsuccessful due to a lack of patient awareness on the importance of treatment adherence (12).

European guidelines for the management of cancer pain, developed by the European Society for Medical Oncology (ESMO) (1), highlight that patients should be empowered and encouraged to openly discuss any suffering or adverse events and the efficacy of their treatment with their clinician. Clinicians should involve the patients in their pain management to improve pain relief through patient understanding and treatment assessment and prescribing (1). Regular patient self-reporting of pain intensity using validated assessment tools, such as the Numerical Rating Scale (NRS), is an essential step towards effective and individualized treatment. Moreover, patients should be appropriately informed about the treatment to be used, especially with the use of opioids (1).

The ESMO guidelines provide precise treatment recommendations for BTcP following a specific assessment of a BTcP episode (1): immediate-release opioids are recommended for BTcP that is opioid-responsive and for which background cancer pain management has been optimized; transmucosal fentanyl formulations for unpredictable and rapid-onset BTcP; and standard, normal-release oral opioids that include a slow-onset BTcP or a preemptive administration of oral opioids 30 min before a predictable BTcP episode triggered by known events. The current ESMO recommendation is to use fentanyl only for patients receiving doses of oral morphine equivalent to at least 60 mg, as this product has been tested in opioid-tolerant patients (13–17). Prescribing decisions need to be based on clinician understanding and experience, product cost and availability, individual patient needs and wishes, and the ability of patients or caregivers to administer the medication (18).

Increasing adherence to ESMO guidelines could be the key to clarifying to the oncology community the gain in patient assessment and management, and to patients the importance of the best treatment for their BTcP. The present study was designed to assess the percentage of patients treated according to the ESMO guidelines (1) for BTcP management in Europe and the impact of adhering to these treatment guidelines on patients' pain relief and quality of life (QoL).

## 2 Materials and methods

### 2.1 Study population and design

This was a prospective, observational, international, multicenter study, providing insights into BTcP management across four European countries: Italy, Spain, Poland, and Czech Republic. The study was carried out from 11 August 2020 (first patient in) to 28 September 2021 (last patient out).

At enrollment into the study, adult patients (>18 years) had to have a diagnosis of locally advanced or recurrent metastatic cancer (histologic or cytologic diagnosis) with a life expectancy of >3 months, an Eastern Cooperative Oncology Group (ECOG) performance status with a score of ≤2, and a diagnosis of BTcP as assessed by the investigator. Moreover, included patients had to be opioid-tolerant, receiving doses of oral morphine of at least 60 mg. Patients were excluded from the study if they had a previous or current history of a clinically significant neurological or psychiatric disorder and/or any current substance abuse or dependence that, according to the investigator's judgment, could impair the study results and if they had a medical condition or situation complicating the collection of data. Patients who had been taking antidepressants and/or drugs acting on pain and who took them on a regular basis during the observation period could be enrolled in the study.

A total of 19 investigational sites enrolled patients. Site investigators were duly selected using a study specific feasibility questionnaire and after that they underwent a pre-study visit to better assess their experience and capabilities; clinical experience in the recognition of BTcP (based on its principal characteristics: high intensity, short time interval between onset and peak intensity, short duration, potential recurrence over 24 h and non-responsiveness to standard analgesic regimes) and its management (knowledge and use of ESMO Clinical Practice guidelines) were taken into main account. In this observational study, BTcP was diagnosed by physician at study inclusion using either the APM algorithm (47.8% of adherent patients) or the clinical experience (52.2% of adherent patients). Once enrolled, all patients were followed for up to 28 days (4 weeks). The follow-up alternated between on-site visits (screening at week 0, visit 1 at week 2 and visit 2 at week 4) and remote visits (telephone contact 1 at week 1 and telephone contact 2 at week 3). BTcP assessment, together with the other study assessments (i.e., Charlson Comorbidity Index, Eastern Cooperative Oncology Group, check of oncological status/concomitant treatments/adverse events, and patient's diary check), were performed at all on-site visits. All patients responded to the Adapted Assessment Tool-BAT and EORTC QLQ-C30 questionnaires at all planned visits, while the PGIC (Patient Global Impression of Change) was assessed only at on-site visits.

The study was conducted in accordance with the Declaration of Helsinki, Ethical Principles for Medical Research Involving Human Patients, applicable Good Clinical Practice principles (19) and Good Pharmacoepidemiology Practice (20). Notification in writing of ethical approval was obtained from the Institutional Ethics Committee and Regulatory Authority before study initiation, as applicable. All patients signed the informed consent form.

### 2.2 Outcomes

The primary endpoint was the percentage of adherent vs. (vs.) non-adherent patients to BTcP treatment according to the ESMO 2018 guidelines (1), in the four weeks of observation (visit 2).

Patients were considered adherent to ESMO guidelines (1) if the following criteria were met throughout the observation period (4 weeks): (1) if patients used immediate-release opioids for BTcP, were opioid-responsive, and their background pain management had been optimized; (2) if patients used transmucosal fentanyl formulations for unpredictable and rapid-onset BTcP; and (3) if patients used standard normal-release oral opioids that included a preemptive administration of oral opioids approximately 30 min before a predictable BTcP triggered by known events. According to ESMO guidelines (1), an optimized background management incorporated primary antitumor treatments, interventional analgesic therapy and non-invasive techniques (psychological and rehabilitative interventions).

BTcP assessment by the APM algorithm (9), BTcP assessment by the adapted Assessment Tool-BAT (11), patients' QoL, and patients' global impression of clinical condition were also evaluated.

QoL was assessed by the European Organization for Research and Treatment of Cancer (EORTC) Quality of Life Questionnaire of Cancer Patients (QLQ-C30) and patients' global impression of clinical condition was assessed by the Patient Global Impression of Change (PGIC). PGIC is a 7-point scale, from very much improved (1) to very much worse (7), with 4 meaning no change.

### 2.3 Statistical analysis

Statistical methods were mainly descriptive or focused on the confidence interval (CI) estimation. All variables were analyzed descriptively with appropriate statistical methods: categorical variables by frequency tables (absolute and relative frequencies) and continuous variables by sample statistics [i.e., mean, standard deviation (SD), 95% CI of the mean, minimum, median, quartiles (Q1; Q3) and maximum]. SAS® version 9.4 was used to analyze the data sets.

## 3 Results

### 3.1 Patient characteristics

Study duration was approximately 13 months. A total of 131 patients were enrolled in the study and 127 patients were included in the evaluable set population (2 patients were screening failures, and 2 patients were excluded because their informed consent forms and patient charts were lost with no possibility to perform adequate clinical monitoring checks). Although all 127 patients fulfilled the inclusion criteria, 37 patients were excluded from the evaluable set population because it was not possible to establish their adherence to the ESMO guidelines: BTcP treatment was not reported in 4 patients, the observational period was less than 4 weeks in 17 patients, background pain treatment was not reported in 11 patients, and BTcP episodes were not reported during the study period in 5 patients (Table 1). As such, 90 (100%) patients included in the evaluation set population were determined adherent to the ESMO guidelines and analyzed for BTcP management and the impact of adhering to ESMO guidelines on their QoL.

**Table 1:** Patients disposition.

Patients enrolled, *n* (%)	131 (100)
Patients included in the evaluable set population^a^, *n* (%)	
Yes	127 (96.9)
No	4 (3.1)
Reason	
Screening failure	2 (50)
Loss of informed consent form and patient chart	2 (50)
Patients adhering to ESMO guidelines	
Yes	90 (70.9)
No	37 (29.1)
Reason	
BTcP episode equal to 0	3 (8.1%)
BTcP episode not reported	2 (5.4%)
BTcP treatment not reported	4 (10.8%)
Background pain treatment not reported	11 (29.7%)
Less than 4 weeks observation	17 (45.9%)

BTcP, breakthrough cancer pain; ESMO, European Society for Medical Oncology.

^a^ All patients who fulfil the inclusion/exclusion criteria.

Adherent patients split roughly evenly in terms of sex (51.1% female) and were a mean (SD) age of 66.4 (11.8) years. Patients had a median (Q1–Q3) Charlson Comorbidity Index (CCI) of 6.0 (3.0–8.0) (Table 2), ECOG performance score of 1.0 (1.0–2.0), and NRS of 4.5 (3.0–6.0). A total of 69 (76.7%) of adherent patients reported any medical history still present at inclusion in the study. Among these patients, 47.8% had hypertension. Moreover, a total of 92 locations of primary tumor were reported at week 0, located mainly in the colon and rectum (15.2% of locations) and the lung (15.2% of locations). The 64.7% of patients had a metastatic disease at study entry; metastases were located mainly in the bone (39.2%), lung (20.7%) and liver (14.1%). A total of 52.2% of patients were under chemotherapy treatment.

**Table 2:** Baseline characteristics of adherent patients.

	*N* = 90
Age (years), mean (SD)	66.4 (11.8)
Female, *n* (%)	46 (51.1)
Country of provenience, *n* (%)	
Italy	22 (24.4)
Spain	10 (11.1)
Poland	57 (63.3)
Czech Republic	1 (1.1)
CCI score, median (Q1–Q3)	6.0 (3.0–8.0)
ECOG score, median (Q1–Q3)	1.0 (1.0–2.0)
NRS score, median (Q1–Q3)	4.5 (3.0–6.0)
Medical history (still present), *n* (%)^a^	*N* = 69
Hypertension	33 (47.8)
Diabetes mellitus	12 (17.4)
Atrial fibrillation	7 (10.1)
Hypercholesterolemia	7 (10.1)
Myocardial ischemia	7 (10.1)
Oncological treatment^a^	
Surgery, *n* (%)	27 (30.0)
Radiotherapy, *n* (%)	33 (36.7)
Chemotherapy, *n* (%)	47 (52.2)
Oncological status (primary tumor locations)	*N* = 92
Location of primary tumor	
Colon and rectal cancer, *n* (%)	14 (15.2)
Lung cancer, *n* (%)	14 (15.2)
Breast cancer, *n* (%)	11 (12.0)
Head and neck, *n* (%)	11 (12.0)
Pancreatic cancer, *n* (%)	6 (6.5)
Stomach, *n* (%)	6 (6.5)
Prostate cancer, *n* (%)	4 (4.3)
Bladder cancer, *n* (%)	4 (4.3)
Kidney cancer, *n* (%)	3 (3.3)
Ovarian cancer, *n* (%)	3 (3.3)
Endometrial cancer, *n* (%)	2 (2.2)
Sarcomas and malignancies of the bone, *n* (%)	2 (2.2)
Liver cancer, *n* (%)	1 (1.1)
Cervical cancer, *n* (%)	1 (1.1)
Hematologic malignancies, *n* (%)	1 (1.1)
Thyroid cancer, *n* (%)	1 (1.1)
Other^b^, *n* (%)	8 (8.74)
Metastasis	
Primary tumors spread to other parts of the body, *n* (%)	62 (67.4)
Bone metastasis, *n* (%)	36 (39.1)
Lung metastasis, *n* (%)	19 (20.7)
Liver metastasis, *n* (%)	13 (14.1)

BTcP, breakthrough cancer pain; CCI, Charlson Comorbidity Index; ECOG, Eastern Cooperative Oncology Group; NRS, numerical rating scale; Q1, 25th percentile; Q3, 75th percentile; SD, standard deviation.

^a^ The data should be read with caution since a patient can be repeated more than once in each group.

^b^ Including tonsils cancer, vulva cancer, oral cancer, tongue cancer, mouth cancer, cutaneous squamous cell carcinoma, cervix cancer and testicular, prostate and rectum cancer.

Overall, 84.4% of adherent patients had concomitant treatments (68.4% of them for alimentary tract and metabolism) and 16.7% of adherent patients took non-pharmacological treatments (60.0% of them underwent psychotherapy) (Table 3). A total of 73.3% of patients received rapid-onset opioids (ROOs). All adherent patients received background pain treatment, mainly fentanyl (51.1% of patients) and buprenorphine (27.8% of patients).

**Table 3:** Patients’ previous and concomitant treatments description.

BTcP category (adherent), *n* (%)	*N* = 90
ROO	66 (73.3%)
Normal-release opioids	10 (11.1%)
ROO + Normal-release opioids	14 (15.6%)
Background pain treatment category (adherent)^a^, *n* (%)	*N* = 90
Morphine	10 (11.1%)
Oxycodone	18 (20.0%)
Oxycodone and naloxone	13 (14.4%)
Fentanyl	46 (51.1%)
Buprenorphine	25 (27.8%)
Oxycodone and paracetamol	1 (1.1%)
Tramadol	1 (1.1%)
Tapentadol	5 (5.6%)
Cannabinoids, includes nabiximols^b^	2 (2.2%)
Methadone	3 (3.3%)
Patients with previous treatments, *n* (%)^c^	*N* = 17
Antiinfectives for systemic use	7 (41.2)
Alimentary tract and metabolism	6 (35.3)
Nervous system	6 (35.3)
Antibacterials for systemic use	5 (29.4)
Patients with concomitant treatments, *n* (%)^c^	*N* = 76
Alimentary tract and metabolism	52 (68.4)
Cardiovascular system	48 (63.2)
Blood and blood forming organs	30 (39.5)
Patients with non-pharmacological treatments, *n* (%)^c^	*N* = 15
Psychotherapy	9 (60.0)
Patients with background pain treatments, *n* (%)^c^	*N* = 90
Analgesics	90 (100.0)
Antiepileptics	40 (44.4)
Patients with BTcP treatments, *n* (%)^c^	*N* = 90
Analgesics	90 (100.0)

BTcP, breakthrough cancer pain; ROO, rapid-onset opioid.

^a^ This data should be read with caution because a patient can be repeated more than once in each category.

^b^ Always in combination with opioids.

^c^ The data should be read with caution since a patient can be repeated more than once in each group.

### 3.2 BTcP assessment

**Figure 1:**
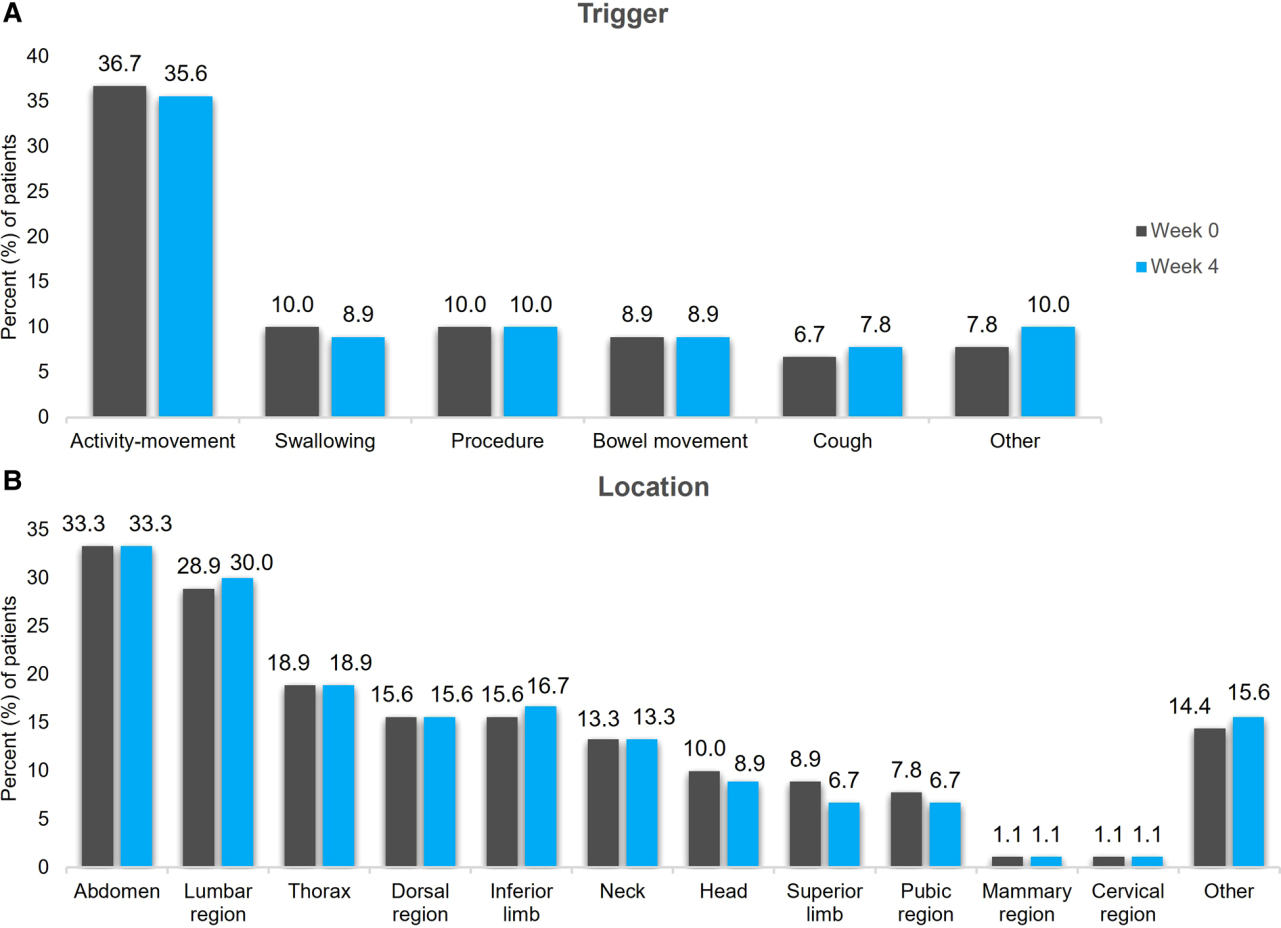
BTcP trigger (**A**) and location (**B**) at week 0 and week 4. BTcP, breakthrough cancer pain.

BTcP assessment at week 0 and week 4 is presented in Table 4. All patients received treatment for BTcP at week 0 and 97.8% were treated at week 4. BTcP was diagnosed either by the APM algorithm in 47.8% of patients or by clinical experience in 52.2% of patients at week 0. At week 0, BTcP was spontaneous or idiopathic in 45.6% of patients, and the origin of pain was somatic nociceptive in 37.8% of patients. BTcP was predictable in 47.8% of patients. The mean number of daily episodes ranged from 1 to 8 at week 4 vs. 3–56 at week 0. The time to maximum pain intensity (onset time) was 3–15 min in 52.2% of patients and longer than 15 min in 36.7% of patients at week 0. Similar results were observed at week 4. Moreover, the main BTcP trigger was activity/movement both at week 0 and at week 4 in 36.7% and 35.6% of patients, respectively (Figure 1A). The main BTcP locations were the abdomen in 33.3% of patients both at week 0 and week 4, and the lumbar region in 28.9% of patients at week 0 and 30.0% of patients at week 4 (Figure 1B).

**Table 4:** BTcP assessment in adherent patients at week 0 and week 4.

	Week 0 (*N* = 90)	Week 4 (*N* = 90)
Diagnosis of BTcP		
APM algorithm, *n* (%)	43 (47.8)	45 (50.0)
Clinical experience, *n* (%)	47 (52.2)	43 (47.8)
Type of BTcP		
Spontaneous/idiopathic, *n* (%)	41 (45.6)	40 (44.4)
Incident/precipitated volitional, *n* (%)	30 (33.3)	32 (35.6)
Incident/precipitated non-volitional, *n* (%)	6 (6.7)	4 (4.4)
Incident/precipitated procedural, *n* (%)	13 (14.4)	12 (13.3)
Quality of pain		
Somatic nociceptive, *n* (%)	34 (37.8)	35 (38.9)
Visceral nociceptive, *n* (%)	11 (12.2)	10 (11.1)
Neuropathic, *n* (%)	20 (22.2)	20 (22.2)
Unspecified, *n* (%)	25 (27.8)	23 (25.6)
Frequency (days)		
Patients with BTcP episodes, *n*/min-max episodes *N*	90/1.0–8.0	88/0.0–29.0
Frequency (weeks)		
Patients with BTcP episodes, *n*/min-max episodes *N*	90/3.0–56.0	88/0.0–56.0
Predictability		
Yes, *n* (%)	43 (47.8)	42 (46.7)
No, *n* (%)	47 (52.2)	46 (51.1)
Onset time		
0–3 min, *n* (%)	9 (10.0)	12 (13.3)
3–15 min, *n* (%)	47 (52.2)	45 (50.0)
>15 min, *n* (%)	33 (36.7)	30 (33.3)
Other, *n* (%)	1 (1.1)	1 (1.1)
BTcP treated		
Yes, *n* (%)	90 (100.0)	88 (97.8)

APM, Association for Palliative Medicine; BTcP, breakthrough cancer pain.

### 3.3 BTcP assessment using the adapted assessment tool-BAT

BTcP assessment using the adapted Assessment Tool-BAT at week 0 and week 4 is presented in Table 5. A total of 44.4% of patients indicated that a typical BTcP episode lasted 5–15 min at week 0. A trend towards a decrease in patients reporting durations of 30–60 min (14.4% of patients at week 0 vs. 4.4% of patients at week 4) and increase in patients reporting durations of 15–30 min (18.9% of patients at week 0 vs. 30.0% of patients at week 4) was observed. Patients characterized the severity of a typical BTcP episode with a mean (95% CI) of 7.3 (7.0; 7.6) at week 0 and 6.2 (5.8; 6.6) at week 4 [scale ranges from 0 (no pain) to 10 (pain as bad as you can imagine)]. Distress caused by BTcP was reported with a mean (95% CI) value of 7.5 (7.1; 7.8) at week 0 and 6.1 (5.7; 6.6) at week 4 [scale ranges from 0 (not at all) to 10 (very much)]. Concerning how much BTcP stopped patients from living a normal life [ranging from 0 (not at all) to 10 (very much)], a mean (95% CI) value of 6.8 (6.4; 7.2) was reported at week 0 and of 6.1 (5.6; 6.5) at week 4. Patients assessed the effectiveness of the painkiller they normally used for BTcP with a mean (95% CI) value of 6.6 (6.1; 7.1) at week 0 and of 7.4 (7.0; 7.8) at week 4 [scale ranges from 0 (not at all effective) to 10 (completely effective)]. A total of 32.2% of patients indicated that the painkiller they used for BTcP had a meaningful effect after 0–10 min and 28.9% of patients after 10–20 min at week 0. At week 4, 36.7% of patients indicated that the painkiller they used for BTcP had a meaningful effect after 0–10 min and 36.7% of patients after 10–20 min.

**Table 5:** BTcP assessment by the adapted assessment tool-BAT in adherent patients at weeks 0 and 4.

	Week 0	Week 4
How long does a typical episode of BTcP last?	*N* = 90	*N* = 90
<5 min, *n* (%)	6 (6.7)	5 (5.6)
5–15 min, *n* (%)	40 (44.4)	41 (45.6)
15–30 min, *n* (%)	17 (18.9)	27 (30.0)
30–60 min, *n* (%)	13 (14.4)	4 (4.4)
>60 min, *n* (%)	14 (15.6)	11 (12.2)
How severe is a typical episode of BTcP?	*N* = 90	*N* = 88
Mean (95% CI)	7.3 (7.0; 7.6)	6.2 (5.8; 6.6)
How much does the BTcP distress you?	*N* = 90	*N* = 88
Mean (95% CI)	7.5 (7.1; 7.8)	6.1 (5.7; 6.6)
How much does the BTcP stop you from living a normal life?	*N* = 90	*N* = 88
Mean (95% CI)	6.8 (6.4; 7.2)	6.1 (5.6; 6.5)
How effective is the painkiller that you usually take for your BTcP?	*N* = 89	*N* = 88
Mean (95% CI)	6.6 (6.1; 7.1)	7.4 (7.0; 7.8)
How long does the painkiller for your BTcP take to have a meaningful effect? (Week 0)	*N* = 90	*N* = 90
No effect, *n* (%)	2 (2.2)	–
0–10 min, *n* (%)	29 (32.2)	33 (36.7)
10–20 min, *n* (%)	26 (28.9)	33 (36.7)
20–30 min, *n* (%)	18 (20.0)	12 (13.3)
>30 min, *n* (%)	14 (15.6)	10 (11.1)

BTcP, breakthrough cancer pain; CI, confidence interval; SD, standard deviation.

### 3.4 Quality of life of patients with BTcP

**Figure 2:**
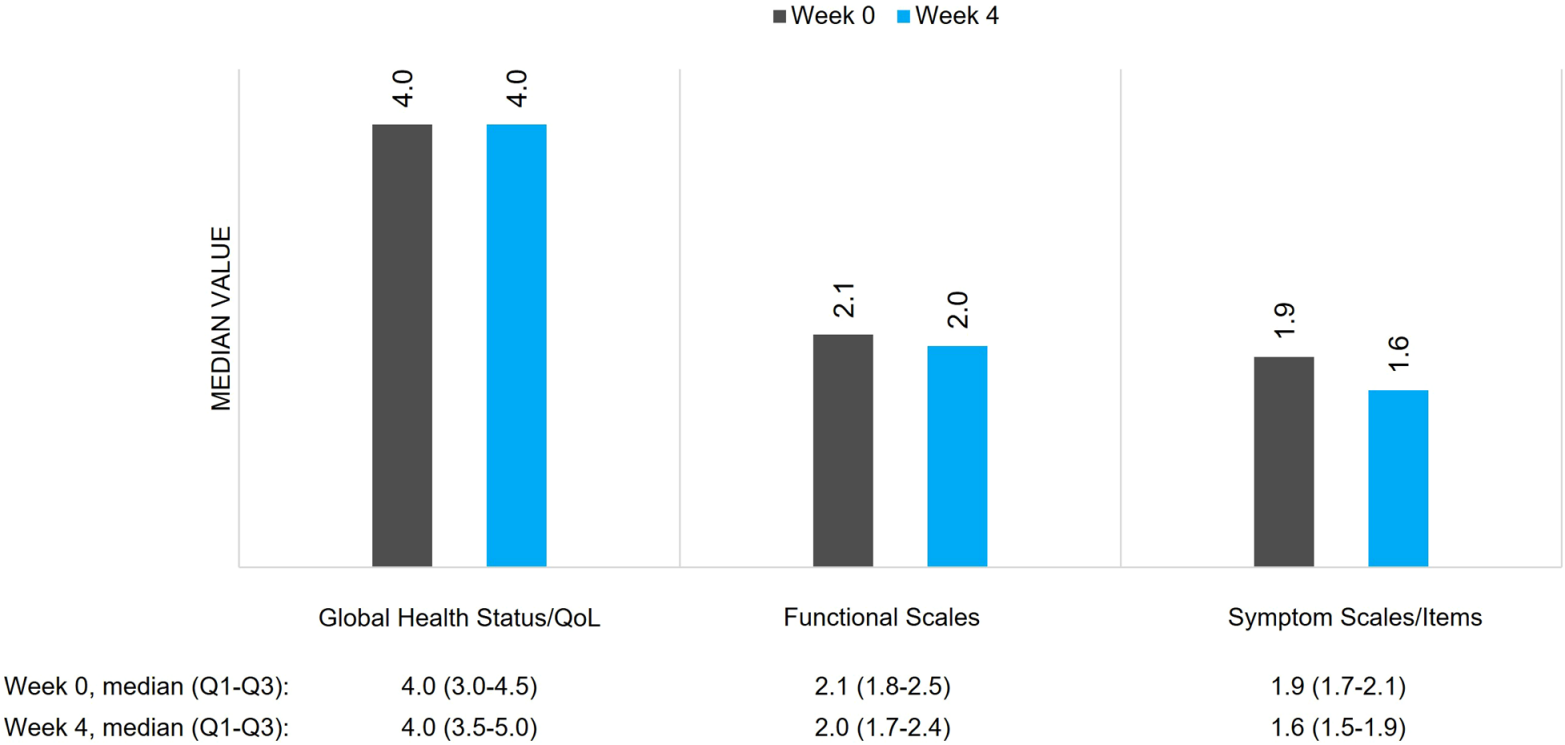
QoL of patients with BTcP assessed by EORTC QLQ-C30. BTcP, breakthrough cancer pain; QoL, quality of life; EORTC QLQ, European Organization for Research and Treatment of Cancer Quality of Life Questionnaire.

QoL was assessed by the EORTC QLQ-C30 questionnaire using three scales: global health status, and functional and symptoms scales. Regarding global health status [range from 1 (very poor) to 7 (excellent)], a median (Q1–Q3) value of 4.0 (3.0–4.5) was reported by patients at week 0 (Figure 2). The median (Q1–Q3) value for functional scales [range from 1 (not at all) to 4 (very much)] was 2.1 (1.8–2.5), whereas the median (Q1–Q3) value for symptom scales [range from 1 (not at all) to 4 (very much)] was 1.9 (1.7–2.1), both at week 0. Similar values were reported at week 4 (Figure 2). The median (Q1–Q3) PGIC of clinical condition was 4.0 (4.0–4.0) (no change) at week 0 (*N* = 89) and 3.0 (2.0–3.0) (minimal improvement) at week 4 (*N* = 88).

## 4 Discussion

ESMO guidelines offer recommendations on BTcP treatment, and increased guidelines compliance should improve assessment and management of BTcP in cancer patients (21). Therefore, this study aimed to assess the adherence to ESMO guidelines for BTcP management and the impact of adherence on patients' QoL.

Adherence to ESMO guidelines (1) was defined as compliance with treatment guidelines for BTcP throughout the observation period (4 weeks). Following these criteria, adherence in our study was 100% as all 90 patients in our sample adhered to the ESMO guidelines. This level of adherence was higher than expected considering previous studies in which the implementation of guidelines for BTcP was assessed. In one study carried out in Spain, it was found that local clinical practice guidelines for the treatment of BTcP were the most known (97% of clinicians), followed by ESMO guidelines (63% of clinicians); a total of 85% of the respondents reported following BTcP management recommendations, and 86% of these relied on local guidelines. Adherence to BTcP treatment recommendations was high, ranging from 76% to 92% (22). In contrast, a survey conducted in 180 Korean clinicians concluded an overall lack of adherence to the guidelines, with a surprising 40% of patients not receiving any prescription for BTcP (21).

In the current study, a total of 37 patients were excluded from the evaluable set population because it was impossible to establish their adherence to ESMO guidelines. In 20 of them, there was missing information about background or BTcP, thus making any evaluation impossible; the other 17 patients had an observation period of less than 4 weeks. It is possible that in the case of these patients, missing data could be due to a lack of adherence since patients and clinicians may not have complied with ESMO guidelines. Thus, in a worst-case scenario the compliance could drop to 70%.

Following the ESMO guidelines, clinicians should involve patients in BTcP pain management (1). Patients' adherence to pain treatment is crucial to obtain the best control of this symptom. Additionally, adherence to guidelines by the clinician allows patients the highest probability of pain control. The high adherence observed in our study could be because clinicians were more adherent to ESMO guidelines precisely because they participated in the study and knew that the primary objective was the rate of adherent patients. Thus, reinforcing the need to encourage clinicians to comply with ESMO guidelines. Indeed, the importance of educating clinicians in this regard was previously reported (23). Also, it should be considered that patients were closely followed up during the study period, with weekly visits or phone contacts. This level of close monitoring of such a difficult-to-treat symptom and a proactive approach could be crucial to a tailored and more effective treatment of pain. Previous experiences with weekly nurse monitoring and patient's empowerment demonstrated improvement of several symptoms in cancer patients, of which pain was one (24).

Moreover, the correct diagnosis of BTcP and the knowledge of a patient's symptoms leads to appropriate and individualized treatment for each patient. Although the APM algorithm for the diagnosis of BTcP is widely used in clinical practice, only half of the patients were diagnosed using this tool. The other half was diagnosed by the oncologist's clinical experience. Therefore, proper knowledge of BTcP symptoms and patients' characteristics improves the management of pain. In our sample, BTcP was mainly idiopathic (46% of patients), and the origin of pain was mainly somatic nociceptive (38% of patients). The current definition of BTcP indicates that pain episodes are usually severe (1), and in our study, BTcP episodes were characterized by patients as moderate to severe at the beginning of the study, suggesting that patients were well diagnosed. Moreover, the fact that 17% of adherent patients took non-pharmacological treatments, mainly psychotherapy, suggests the need for a comprehensive and holistic approach to pain management (25, 26).

The number of daily episodes reported at inclusion varied from 1 to 8, similar to that reported in other studies (3, 4). Some studies reported a frequency ranging from 3 to 6 daily episodes and others from 1 to 10 daily episodes (27). In this regard, a correlation between a higher number of BTcP episodes with age, head and neck cancer, higher Karnofsky levels, background pain intensity, predictable BTcP, and fast onset was observed (27). On the other hand, in our study, a typical BTcP episode lasted mainly 5–15 min, although some patients reported a duration of 30–60 min or >60 min. Previous surveys reported that the mean duration of untreated BTcP episodes was 30–60 min (3, 28, 29). However, the duration of BTcP episodes decreased during the 4 weeks of the study, possibly witnessing that with an adequate management of BTcP one may also decrease the duration of the pain episodes. Furthermore, the level of distress caused by BTcP tended to decrease along the study period, suggesting a possible learning by patients to manage BTcP and/or a more careful approach by the clinician.

Regarding BTcP treatment, patients reported a high level of opioid effectiveness. In that sense, a recent study reported that 71% of patients were satisfied with their BTcP treatment and that the use of opioid drugs correlated statistically to more satisfaction (vs. none or other therapies) (30). Previously, it was also demonstrated that the level of satisfaction was significantly associated with the use of ROOs (1, 29, 31, 32), which is consistent with our observation that ROOs were used by 73% of adherent patients and a meaningful effect was observed after 0–20 min.

Considering QoL, results from the BEST study showed that an adequate management of BTcP can improve a patient's health-related QoL assessed by EORTC QLQ-C15-PAL, Pittsburgh Sleep Quality Index, and the Edmonton Symptom Assessment System, after 28 days of observation (33). In our study, QoL assessed by the EORTC QLQ-C30 seemed to have not improved along the observational period. It should be noted that one reason for the lack of QoL improvement was the inclusion of patients in different phases of their cancer treatment journey, thus not all patients were captured in the same oncological setting. Also, the short duration of the observational period (4 weeks) could be insufficient to observe substantial changes in QoL domains. However, although the QoL seemed to not improve during the observational period, patients’ global impression (PGIC) of BTcP showed a tendency to improve and the pain severity and the level of distress decreased, showing a relief of the patients' pain.

One of the limitations of our study could be that in about half of the patients the BTcP was diagnosed through physician clinical experience, meaning not using a diagnostically validated or disease-specific instruments, potentially leading to a not truly correct diagnosis. In addition, another limitation of this study concerns the absence of a formal sample size calculation due to the lack of data related to ESMO 2018 guidelines adherence. We are aware that the present study does not have enough power to assess the impact of adhering to the ESMO guidelines and that the statistical methods are only descriptive, with no insights on association between adherence to the ESMO guidelines and impact on pain relief and quality of life, but the study is however very informative of pain control and management in a real word setting. An in-depth description of adherent patients shows that good management of the disease can bring benefits. Furthermore, although the number of 300 patients was judged as substantially adequate to describe a population of patients with BTcP to assess the consistency with the recognized guidelines of treatment, a total of 131 patients was finally enrolled. This was due to the very low enrolment rate during the COVID-19 pandemic.

Due to the restrictiveness of the inclusion criteria, the external validity of the study results may be limited beyond outpatients with ECOG performance status from 0 to 2. Although a lower number of patients than planned were included, it was sufficient to assess the objectives. On the other hand, to avoid selection bias, recruitment was consecutive across different centers and countries. Even though more than half of the patients in this study were enrolled in Poland (63% of patients), the compliance to treatment guidelines was high in all the involved countries, reflecting a high quality of clinical practice. Moreover, representative sites were selected within this country to reflect the full range of settings managing BTcP patients.

Nevertheless, we strongly think our trial demonstrates the need to raise awareness about the BTcP treatment and its correct identification. Even in a well selected group of physicians, having experience in the management of cancer patients with pain, the definition of BTcP has been relaxed, in that a few patients indicated a high number of BTcP episodes per day, which is not often consistent with the definition of BTcP itself.

## 5 Conclusion

In conclusion, if a patient is properly diagnosed and educated to recognize BTcP episodes and closely follow-up by the clinician (i.e., weekly), BTcP can be properly treated, with a possible improvement in management and patients' impression of change. Therefore, clear assessment and strict follow-up of patients by means of clinical visit and phone call could be crucial to comply with ESMO guidelines.

## Data Availability

The raw data supporting the conclusions of this article will be made available by the authors, without undue reservation.

## References

[1] Fallon M Giusti R Aielli F Hoskin P Rolke R Sharma M Management of cancer pain in adult patients: ESMO clinical practice guidelines. Ann Oncol. (2018) 29:iv166–91. 10.1093/annonc/mdy152 30052758

[2] Deandrea S Corli O Consonni D Villani W Greco MT Apolone G. Prevalence of breakthrough cancer pain: a systematic review and a pooled analysis of published literature. J Pain Symptom Manage. (2014) 47(1):57–76. 10.1016/j.jpainsymman.2013.02.015 23796584

[3] Davies A Zeppetellal G Andersenl S Damkierl A Vejlgaardl T Nauckl F Multi-centre European study of breakthrough cancer pain: pain characteristics and patient perceptions of current and potential management strategies. Eur J Pain. (2011) 15(7):756–63. 10.1016/j.ejpain.2010.12.004 21251860

[4] Portenoy RK Payne D Jacobsen P. Breakthrough pain: characteristics and impact in patients with cancer pain. Pain. (1999) 81(1):129–34. 10.1016/S0304-3959(99)00006-8 10353500

[5] American Pain Foundation. Breakthrough cancer pain: mending the break in the Continuum of care. J Pain Palliat Care Pharmacother. (2011) 25(3):252–64. 10.3109/15360288.2011.599920 21882979

[6] Scharpf J Karnell LH Christensen AJ Funk GF. The role of pain in head and neck cancer recurrence and survivorship. Arch Otolaryngol Head Neck Surg. (2009) 135(8):789. 10.1001/archoto.2009.107 19687400

[7] Caraceni A Martini C Zecca E Portenoy RK, AWorking Group of an IASP Task Force on Cancer Pain. Breakthrough pain characteristics and syndromes in patients with cancer pain. An international survey. Palliat Med. (2004) 18(3):177–83. 10.1191/0269216304pm890oa 15198130

[8] Vellucci R Fanelli G Pannuti R Peruselli C Adamo S Alongi G What to do, and what not to do, when diagnosing and treating breakthrough cancer pain (BTcP): expert opinion. Drugs. (2016) 76(3):315–30. 10.1007/s40265-015-0519-2 26755179 PMC4757619

[9] Davies AN Dickman A Reid C Stevens AM Zeppetella G. The management of cancer-related breakthrough pain: recommendations of a task group of the science committee of the association for palliative medicine of Great Britain and Ireland. Eur J Pain. (2009) 13(4):331–8. 10.1016/j.ejpain.2008.06.014 18707904

[10] Portenoy RK Bruns D Shoemaker B Shoemaker SA. Breakthrough pain in community-dwelling patients with cancer pain and noncancer pain, part 1: prevalence and characteristics. J Opioid Manag. (2010) 6(2):97–108. 10.5055/jom.2010.0009 20481174

[11] Webber K Davies AN Zeppetella G Cowie MR. Development and validation of the breakthrough pain assessment tool (BAT) in cancer patients. J Pain Symptom Manage. (2014) 48(4):619–31. 10.1016/j.jpainsymman.2013.10.026 24766740

[12] Margarit C Antón A Escobar Y Casas A Cruz JJ López R Breakthrough cancer pain—still a challenge. JPR. (2012) 5:559–66. 10.2147/JPR.S36428 PMC3508660 23204865

[13] Mercadante S. Fentanyl buccal tablet for the treatment of cancer-related breakthrough pain. Expert Rev Clin Pharmacol. (2015) 8(1):9–13. 10.1586/17512433.2015.977254 25359295

[14] Rauck R Reynolds L Geach J Bull J Stearns L Scherlis M Efficacy and safety of fentanyl sublingual spray for the treatment of breakthrough cancer pain: a randomized, double-blind, placebo-controlled study. Curr Med Res Opin. (2012) 28(5):859–70. 10.1185/03007995.2012.683111 22480131

[15] Rogríguez D Urrutia G Escobar Y Moya J Murillo M. Efficacy and safety of oral or nasal fentanyl for treatment of breakthrough pain in cancer patients: a systematic review. J Pain Palliat Care Pharmacother. (2015) 29(3):228–46. 10.3109/15360288.2015.1047554 26458018

[16] Shimoyama N Gomyo I Katakami N Okada M Yukitoshi N Ohta E Efficacy and safety of sublingual fentanyl orally disintegrating tablet at doses determined by titration for the treatment of breakthrough pain in Japanese cancer patients: a multicenter, randomized, placebo-controlled, double-blind phase III trial. Int J Clin Oncol. (2015) 20(1):198–206. 10.1007/s10147-014-0697-z 24839047

[17] Slatkin NE Xie F Messina J Segal TJ. Fentanyl buccal tablet for relief of breakthrough pain in opioid-tolerant patients with cancer-related chronic pain. J Support Oncol. (2007) 5(7):327–34. 17708123

[18] Smith H. Considerations in selecting rapid-onset opioids for the management of breakthrough pain. J Pain Res. (2013) 6:189–200. 10.2147/JPR.S40745 23503653 PMC3594916

[19] World Medical Association. WMA Declaration of Helsinki—Ethical Principles for Medical Research Involving Human Subjects. Ferney-Voltaire, France: World Medical Association (2022). Available online at: https://www.wma.net/policies-post/wma-declaration-of-helsinki-ethical-principles-for-medical-research-involving-human-subjects/ (cited April 18, 2023).

[20] International Society for Pharmacoepidemiology. Guidelines for Good Pharmacoepidemiology Practices (GPP). Washington, DC: International Society for Pharmacoepidemiology (2015). Available online at: https://www.pharmacoepi.org/resources/policies/guidelines-08027/ (cited April 18, 2023).

[21] Kim DY Ahn JS Lee KH Kim YC Lee J Kim SY. A nationwide survey of knowledge of and compliance with cancer pain management guidelines by Korean physicians. Cancer Res Treat. (2014) 46(2):131–40. 10.4143/crt.2014.46.2.131 24851104 PMC4022821

[22] López López R Camps Herrero C Khosravi-Shahi P Guillem Porta V Carrato Mena A Garcia-Foncillas J Oncologist’s knowledge and implementation of guidelines for breakthrough cancer pain in Spain: CONOCE study. Clin Transl Oncol. (2018) 20(5):613–8. 10.1007/s12094-017-1756-5 28975575

[23] Bossi P Escobar Y Pea F. Rapid-onset opioids for management of breakthrough cancer pain: considerations for daily practice. Front Pain Res. (2022) 3:893530. 10.3389/fpain.2022.893530 PMC9204512 35721659

[24] Antonuzzo A Ripamonti CI Roila F Sbrana A Galli L Miccinesi G Effectiveness of a phone-based nurse monitoring assessment and intervention for chemotherapy-related toxicity: a randomized multicenter trial. Front Oncol. (2022) 12:925366. 10.3389/fonc.2022.925366 36185306 PMC9520968

[25] Filipponi C Masiero M Pizzoli SFM Grasso R Ferrucci R Pravettoni G. A comprehensive analysis of the cancer chronic pain experience: a narrative review. Cancer Manag Res. (2022) 14:2173–84. 10.2147/CMAR.S355653 35855762 PMC9288227

[26] Kress HG Aldington D Alon E Coaccioli S Collett B Coluzzi F A holistic approach to chronic pain management that involves all stakeholders: change is needed. Curr Med Res Opin. (2015) 31(9):1743–54. 10.1185/03007995.2015.1072088 26172982

[27] Mercadante S Marchetti P Cuomo A Caraceni A Mediati R Vellucci R Factors influencing the clinical presentation of breakthrough pain in cancer patients. Cancers (Basel). (2018) 10(6):175. 10.3390/cancers10060175 29865170 PMC6025469

[28] Hjermstad MJ Kaasa S Caraceni A Loge JH Pedersen T Haugen DF Characteristics of breakthrough cancer pain and its influence on quality of life in an international cohort of patients with cancer. BMJ Support Palliat Care. (2016) 6(3):344–52. 10.1136/bmjspcare-2015-000887 27342412

[29] Mercadante S Marchetti P Cuomo A Caraceni A Mediati RD Mammucari M Breakthrough cancer pain: preliminary data of the Italian oncologic pain multisetting multicentric survey (IOPS-MS). Adv Ther. (2017) 34(1):120–35. 10.1007/s12325-016-0440-4 27873235 PMC5216057

[30] Mazzotta M Filetti M Piras M Mercadante S Marchetti P Giusti R. Patients’ satisfaction with breakthrough cancer pain therapy: a secondary analysis of IOPS-MS study. Cancer Manag Res. (2022) 14:1237–45. 10.2147/CMAR.S353036 35356594 PMC8959622

[31] Giusti R Bossi P Mazzotta M Filetti M Iacono D Marchetti P. The use of fentanyl in pain management in head and neck cancer patients: a narrative review. Br J Pain. (2018) 12(3):155–62. 10.1177/2049463717736787 30057760 PMC6058455

[32] Pointreau Y Bensadoun RJ Bera G Sire C Ruffier A Janoray G Patient satisfaction with fentanyl pectin nasal spray in breakthrough cancer pain management during radiotherapy for head and neck cancer. Patient Prefer Adherence. (2020) 14:859–68. 10.2147/PPA.S246757 32546980 PMC7247611

[33] Cuomo A Cascella M Forte CA Bimonte S Esposito G De Santis S Careful breakthrough cancer pain treatment through rapid-onset transmucosal fentanyl improves the quality of life in cancer patients: results from the BEST multicenter study. J Clin Med. (2020) 9(4):1003. 10.3390/jcm9041003 32252426 PMC7230287

